# Rapid Determination of Isomeric Benzoylpaeoniflorin and Benzoylalbiflorin in Rat Plasma by LC-MS/MS Method

**DOI:** 10.1155/2017/1693464

**Published:** 2017-05-08

**Authors:** Chuanqi Zhou, Xiaoke Wang

**Affiliations:** ^1^Key Laboratory of Molecular Diagnosis and Medicinal Chemistry, Ministry of Education, Hebei University, Baoding 071002, China; ^2^College of Chemistry & Environmental Science, Hebei University, Baoding 071002, China

## Abstract

Benzoylpaeoniflorin (BP) is a potential therapeutic agent against oxidative stress related Alzheimer's disease. In this study, a more rapid, selective, and sensitive liquid chromatography-tandem mass spectrometric (LC-MS/MS) method was developed to determine BP in rat plasma distinguishing with a monoterpene isomer, benzoylalbiflorin (BA). The method showed a linear response from 1 to 1000 ng/mL (*r* > 0.9950). The precision of the interday and intraday ranged from 2.03 to 12.48% and the accuracy values ranged from −8.00 to 10.33%. Each running of the method could be finished in 4 minutes. The LC-MS/MS method was validated for specificity, linearity, precision, accuracy, recovery, and stability and was found to be acceptable for bioanalytical application. Finally, this fully validated method was successfully applied to a pharmacokinetic study in rats following oral administration.

## 1. Introduction

Alzheimer's disease (AD) is a chronic neurodegenerative disease that accounts for 60% to 70% of cases of dementia [1, 2]. Oxidative stress has been generally recognized as a cause of various neurodegenerative disorders including AD [3, 4]. Therefore, diverse antioxidants have been investigated as potential therapeutic agents against oxidative stress related AD. Benzoylpaeoniflorin (BP), a kind of monoterpene glycoside mainly isolated from* Paeonia* species, has been proved on strong antioxidant property in primary cultures of rat cortical cells against H_2_O_2_-induced neurotoxicity [5]. Recent attention has been paid to a role of BP in individual animal because exciting progress in the field of neurotoxicity is emerging through the establishment of experimental implementation of antioxidant activity. However, the quantification of BP in plasma and its role in neurotoxicity is yet to be identified. The goal of our analytical study is to develop a simple, sensitive, accurate, and reproducible analytical method for BP quantification in rat plasma, available for use in the dynamic analysis of the BP concentration profile in animal.

Several kinds of methods have been proposed for the determination of BP, based on HPLC with UV detection or LC-MS/MS [6–9]. In particular, the LC-MS/MS method is expected to be a powerful tool for the quantification of BP in biological fluids, because it generally offers superior selectivity and sensitivity for the detection of low concentration analytes in a complex matrix, such as plasma and tissue extracts [10–13]. However, interfering of monoterpene isomers remains challenging for the identification and quantification of BP from complex matrix, especially for benzoylalbiflorin (BA), a BP isomer with equivalent groups of benzoic acid and glucose and similar structure of aglycone [14]. In previous research, BA coexisted with BP in the methanolic extract of the roots of* Paeonia lactiflora* and resembled BP in most of diagnostic fragmentations or characteristic ions on ESI-MS/MS spectra, when LC-MS/MS method was used [14, 15]. Therefore, new transition pair of the complete removal of BA cross-talking is essential for the selective and robust determination. The investigation of BA will take specific insight into the identification and quantification of BP in rat plasma. In addition, solid-phase extraction (SPE) is not suitable for the high-throughput analysis of many plasma samples and is not considered in our experiments due to time consumption and several steps.

In this study, we have developed a rapid and specific LC-MS/MS method for the determination of BP in rat plasma, which is simple, time-saving, operational, and distinguished from BA. Additionally, method validation according to Chinese Pharmacopoeia (2010 Edition) and statistical evaluation of data will be conducted. If improved, it may also be applied to other administration routes and other animal species or humans.

## 2. Experimental

### 2.1. Chemicals

Puerarin (internal standard (IS),* N* > 98.0% purity) was purchased from J&K Scientific Ltd (Beijing, China).* Paeonia lactiflora* was supplied by Qixin Chinese medicine particles Co. (Hebei, China) and were identified in accordance with each standard stipulated in the Chinese Pharmacopoeia (2010 Edition). BP (99% purity) and BA (99% purity) standards were prepared in our laboratory from* Paeonia lactiflora*. Methanol (HPLC Grade) was purchased from Thermo Fisher Scientific (MA, USA). Ultrapure water was prepared using a Milli-Q Reagent Water System (Millipore, MA, USA) throughout the study.

### 2.2. Preparation of Standard and Quality Controls

Each stock solution of BP, BA, and IS was prepared at 1 mg/mL in methanol. Working standard solutions for calibration and controls were prepared by serial dilution of the stock solution with methanol. The working standard solution for IS (100 ng/mL) was prepared by diluting its stock solution with methanol. Calibration standards were prepared by spiking blank rat plasma with appropriate amounts of the working solutions yielding final concentrations of 1, 2, 5, 10, 50, 100, 500, and 1000 ng/mL for BP and BA. Quality control (QC) samples for BP and BA were prepared by the same way to achieve 1 ng/mL, 2 ng/mL, 500 ng/mL, and 750 ng/mL. All stock and working solutions were stored prior to validation at −20°C and 4°C, respectively, and brought to room temperature before use.

### 2.3. HPLC Conditions

All samples were analyzed by isocratic elution. Chromatographic separation was carried out on Ultimate 3000 with a Hypersil C_18_ column (2.1 × 50 mm, 3 *μ*m; Thermo Fisher Scientific, USA) at a flow rate of 150 *μ*L/min with the temperature maintained at 30°C. The mobile phase was acetonitrile/water (90 : 10, v/v) containing 0.1% formic acid. Each running could be closed in 4 minutes.

### 2.4. Mass Spectrometry Conditions

The LC system was monitored with triple quadrupole tandem mass spectrometry (API 4000, AB Sciex, CA, USA) equipped with an TurboV electrospray ionization (ESI) source for positive and negative mode. Multiple reaction monitoring (MRM) data were acquired and processed with the Analyst software (AB Sciex, MA, USA). The optimized operation parameters of MRM are listed in Table 1.

### 2.5. Method Validation

The selectivity of the method was evaluated by analyzing six lots of blank rat plasma, blank plasma spiked with BP, BA, and IS, and a rat plasma sample after oral administration of BP. Calibration curves were constructed by analyzing spiked calibration samples on three separate days. Peak area ratios of analyte to IS were plotted against analyte concentrations, and standard curves were well fitted to the equations by linear regression in the concentration range of 1–1000 ng/mL. The lower limit of quantification (LLOQ) is the lowest concentration of analyte which can be quantified reliably, with an acceptable accuracy (80–120%) and precision (<20%). The LLOQ is considered as the lowest calibration standard.

Precision and accuracy were assessed by the determination of QC samples in six replicates in three validation days. The precision was reflected by relative standard deviation (RSD) and the accuracy by relative error (RE). The intraday and interday RSD value should not exceed 15% for the QC samples.

The extraction recovery was evaluated by comparing the peak area of extracted QC samples with those of reference QC solutions reconstituted in blank plasma extracts (*n* = 6). To evaluate the matrix effect, blank rat plasma was extracted and then spiked with QC samples. The corresponding peak areas were then compared with those of neat standard solutions at equivalent concentrations, and this peak area ratio is defined as the matrix effect.

Stability of analytes in QC samples was determined by analyzing in triplicate under different conditions: three freeze-thaw cycles were carried out, with samples stored at −20°C for 24 h and thawed to 20°C. Short-term stability was assessed by storing samples at 25°C for 4 h. The long term stability was accessed by storage at −20°C for 30 days. The results were compared with those obtained with freshly prepared QC samples.

### 2.6. Pharmacokinetic Study

Twelve male Wistar rats (250–300 g) were obtained from Laboratory Animal Centre of Hebei University (Baoding, China). They were kept in an environmentally controlled breeding room for 3 days before starting the experiments and fed with standard laboratory food and water and fasted overnight before dosing. All rats were divided into two groups randomly. One group was administered with an oral dose of 19 mg/kg of BP and the other was with the same dose of BA.

Blood samples were collected via the postorbital venous plexus veins into 1.5 mL heparinized polythene tubes at 5,10,15,20,30,40,60,120,240,360,480 and 540 min after intragastric administration. The heparinized blood was immediately centrifuged for 10 min at 4000 rpm/min to yield the plasma, stored at −20°C until analysis.

The pharmacokinetic analysis was used to determine concentration-time profiles of BP and BA in rat plasma after a single intragastric administration. Data of BP and BA concentrations versus time for each rat were analyzed by the DAS software (version 2.0, Mathematical Pharmacology Professional Committee of China, Shanghai, China). BP or BA plasma concentrations at different times were expressed as mean ± SD and the concentration-time curve was plotted. The pharmacokinetic parameters of the BP and BA with the* t*-test were analyzed using SPSS l8.0 statistical software. A value of *p* < 0.05 was considered statistically significant.

## 3. Results and Discussion

### 3.1. Chromatographic Condition and MS Parameter Optimization

To avoid residual signals and inaccuracies, the chromatographic conditions were established in isocratic mode for sample analysis. The peak shapes and MS signals of the analytes were improved by using a mobile phase of acetonitrile/water (90 : 10, v/v) containing formic acid (0.1%, v/v). Each running could be finished in 4 minute.

**Figure 1:**
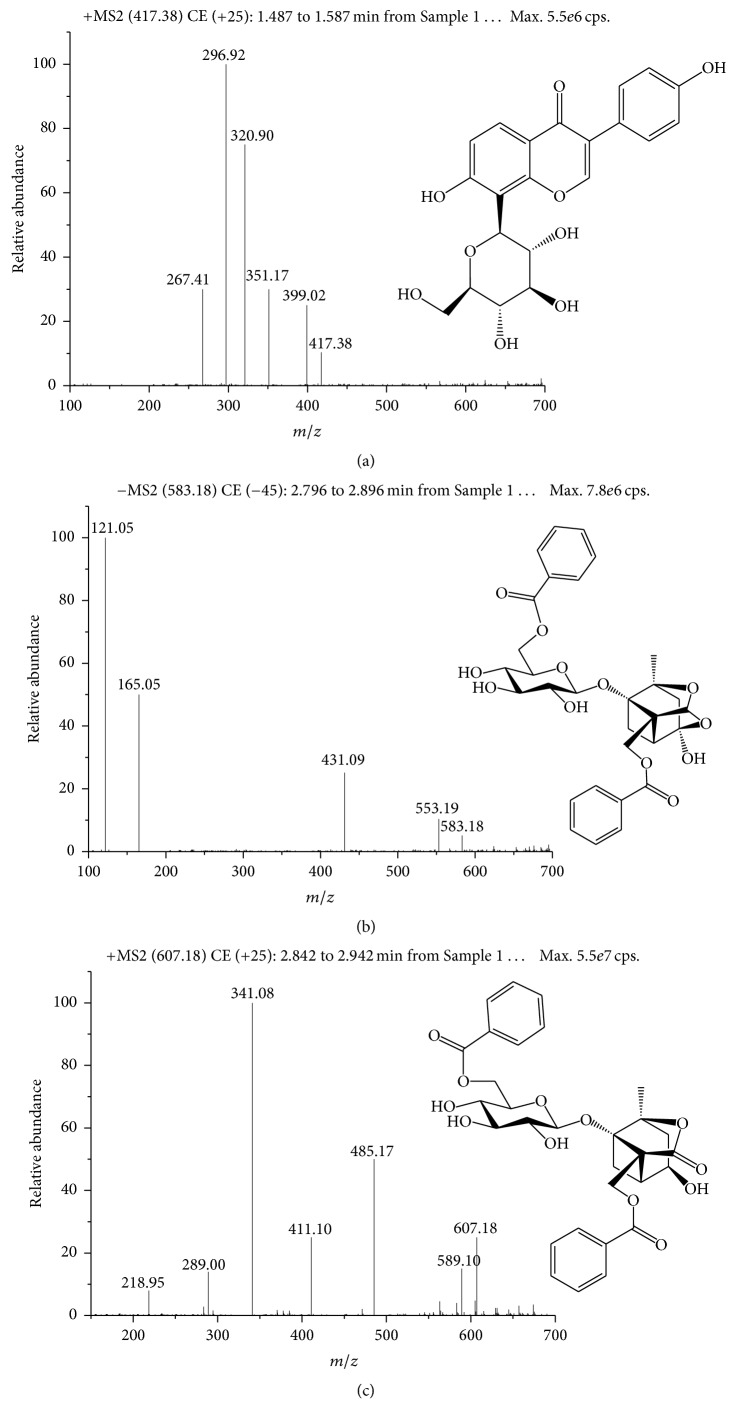
MS/MS spectra and structures of the two analytes and IS: (a) IS, parent ion:* m/z* 417.38; daughter ion:* m/z* 296.90; (b) BP, parent ion:* m/z* 583.18; daughter ion:* m/z* 165.05; (c) BA, parent ion:* m/z* 607.18; daughter ion:* m/z* 589.10.

**Figure 2:**
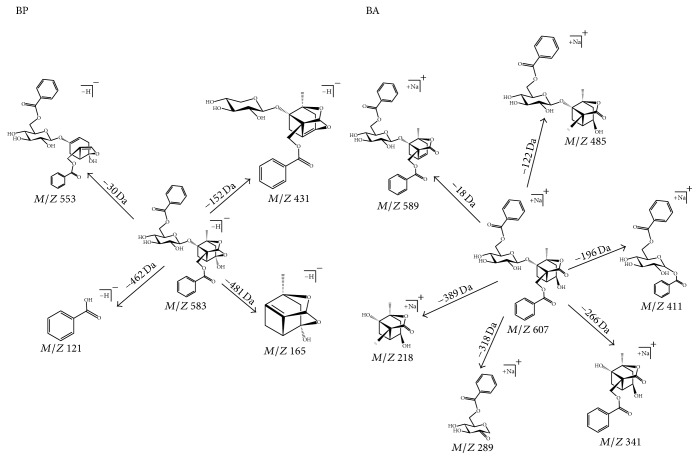
The structure elucidation on BP in negative mode and BA in positive mode.

**Figure 3:**
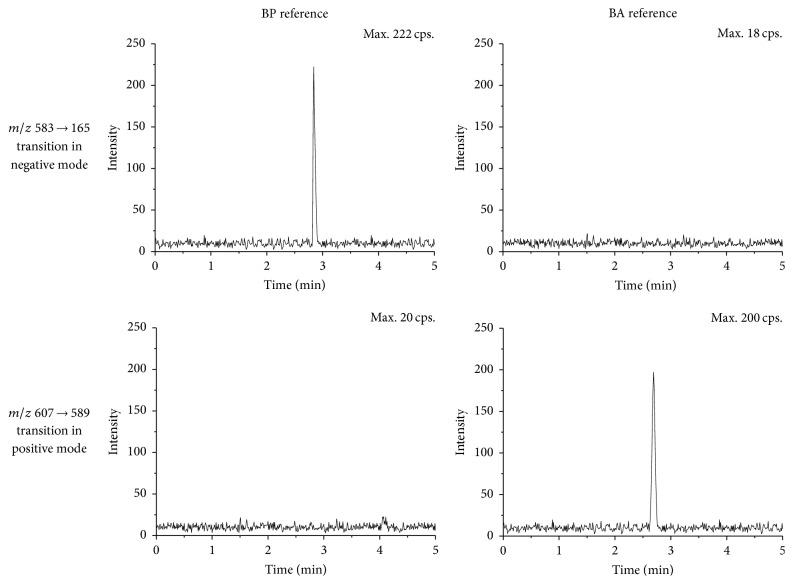
The specificity of* m/z *583 → 165 in negative mode and* m/z *607 → 589 positive mode on BP and BA references.

Owing to lack knowledge about BA, there are still some imperfections in the appraisal of the BP quantification. To distinguish between BP and BA, multiple reaction monitoring (MRM) of the transitions were* m/z *583.18 → *m*/*z* 165.05 for BP in negative mode,* m/z *607.18 → 589.10 for BA, and* m/z *417.38 → 296.90 for IS in positive mode, respectively (Figure 1). The collision energy determining product ion signal intensity was 25 eV for both BP and BA, while 45 eV was used for IS. The optimized MRM parameters are listed in Table 1. During the process of selection on MRM transitions, positive and negative modes were investigated to obtain the optimal transition-pair ions. The specificity of each transition channel was monitored and assessed on BP and BA references. In positive mode, ion* m/z* 607 from BP produced main daughter ions including 485,411,375,341,289 and 218, while ion* m/z* 607 from BA produced main daughter ions including 589,485,411,341,289 and 218. The structure elucidation was shown in Figure 2 and supplementary information (Figure S1 and Figure S3 in Supplementary Material available online at https://doi.org/10.1155/2017/1693464). Obviously, daughter ions of* m/z* 375 in BP and* m/z* 589 in BA should verify the specificity of* m/z *607 → 375 and* m/z *607 → 589 transitions. In negative mode, ion* m/z* 583 from BP produced main daughter ions including 553, 431, 165 and 121, while ion* m/z* 583 from BA produced main daughter ions including 431,195 and 121. The structure elucidation could be seen in Figure 2 and supplementary information (Figure S2 and Figure S4). Daughter ions of* m/z* 553, 165 in BP and* m/z* 195 in BA were collected to verify the specificity of* m/z *583 → 165 and* m/z *583 → 195 transitions. Specificity experiments indicated that ions on* m/z *583 → 553,* m/z *583 → 195 in negative mode, and* m/z *607 → 375 in positive mode caused cross-activity on BP and BA references, which hints that lower relative abundance of particle could influence the specificity on MRM experiments (supplementary information, Figure S5). However, ions on* m/z *583 → 165 in negative mode and* m/z *607 → 589 in positive mode were specific for BP and BA, respectively (Figure 3). Therefore, the above two channels could be used to the following studies.

### 3.2. Method Validation

**Figure 4:**
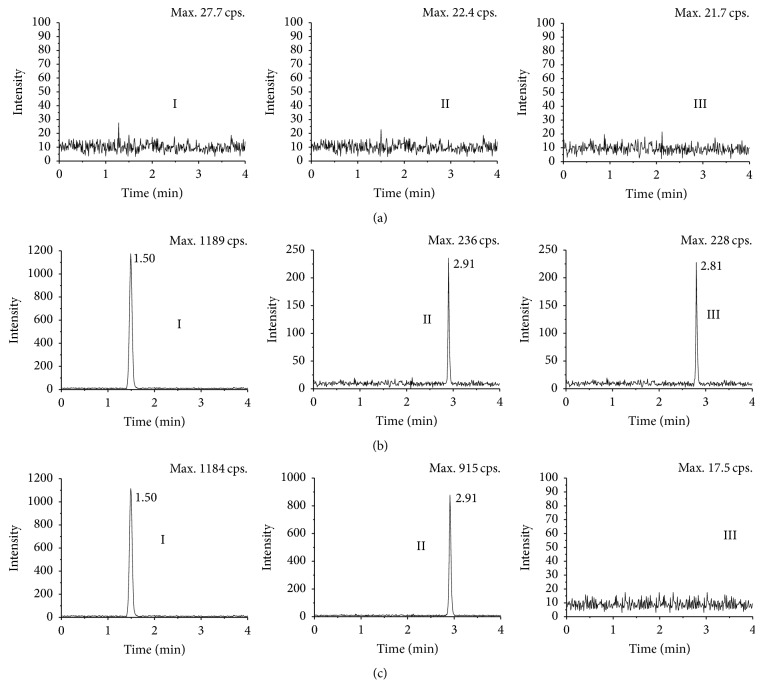
Typical MRM chromatograms of IS, BP, and BA: (a) blank rat plasma; (b) blank rat plasma spiked with IS (I, 100 ng/mL), BP (1 ng/mL), and BA (1 ng/mL); (c) rat plasma sample at 20 min after intragastric administration of BP spiked with IS; Roman numerals: I for IS, II for BP, and III for BA.

The MRM chromatogram of blank plasma, blank plasma spiked with BP, BA, and IS, and samples collected 20 min after intragastric administration with BP are displayed in Figure 4. Although the different retention time between BP and BA is only 0.1 min, excellent peak shapes were exhibited for both analytes and IS, indicating no significant interference from endogenous substances near BP, BA, or IS retention times.

Peak area ratios of BP or BA to IS (*y*) versus analyte concentrations (*x*) were fitted at the concentration range of 1–1000 ng/mL in rats plasma. Typical equations for the calibration curves are listed in Table 2. The correlation coefficients for BP and BA were 0.9998 and 0.9982 (>0.995), with the equations showing good linearity between peak areas ratios and concentrations. Under optimized conditions, The LLOQ for BP and BA were 1.0 and 1.0 ng/mL, respectively.

The precision and accuracy of intra- and interday measurements were evaluated using QC sample. The data obtained for BP and BA (*n* = 6) are summarized in Table 3. Interday precision and accuracy were determined on five consecutive days. The intraday and interday precisions of BP and BA ranged from 2.03 to 12.48%. The accuracies of BP and BA ranged from −8.00 to 10.33%. These findings indicated that precision and accuracy were within the acceptable limits.

The mean recovery rates for BP and BA were from 93 ± 14.29% to 99 ± 6.62% (Table 3). The data demonstrated that the extraction method was consistent and reproducible at different QC levels and suitable for BP and BA. The matrix effects for BP and BA were within 93 ± 14.29% to 99 ± 14.29%. Ion suppression caused by the plasma matrix was negligible.

Stability was determined by analyzing QC samples in triplicate and three different conditions. The results are displayed in Table 4. BP, BA, and IS were stable in plasma at room temperature for 4 h, after three freeze-thaw cycles, and at −20°C for at least one month. These results indicated the method was accurate, reliable, and reproducible.

### 3.3. Pharmacokinetic Study

**Figure 5:**
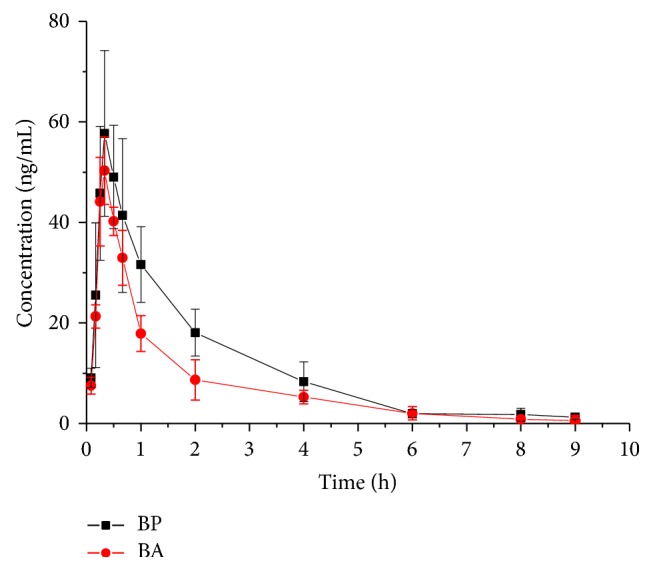
Mean plasma concentration-time profiles of BP and BA in rats after intragastric administration. Data are mean ± SD (*n* = 6).

To further assess the pharmacokinetics of BP in rats, the main pharmacokinetic parameters of BP and BA were compared in Table 5 using a noncompartmental model (Figure 5). *T*_max_ and *t*_1/2_ were 0.35 ± 0.07 and 1.97 ± 0.23 for BP and 0.31 ± 0.03 and 1.47 ± 0.30 for BA which showed no significant differences between BP and BA. However, *C*_max_, AUC_0-*t*_, and MRT_0-∞_ were slightly higher in BP than in BA. To investigate pharmacokinetic profiles of BP and BA, these findings indicated that further researches need focus on the distribution of BP and BA in different tissues.

## 4. Conclusion

This study is the first validated biological analysis of BP in rat plasma distinguishing with BA isomer. This proposed method was successfully applied to pharmacokinetic study of BP and BA, which presented the advantages of high specificity, sensitivity, relatively simple preparation, and high extraction recovery rate. Similar pharmacokinetic curves and parameters were obtained for BP and BA, excluding *C*_max_ and AUC parameters. The pharmacokinetic study showed that BP and BA were quickly eliminated in rats. Therefore, both BP and BA, likely, could play an important role in the pharmacological effects.

## Supplementary Material

Structure elucidations of BP and BA in positive or negative mode.

## Figures and Tables

**Table 1:** MS parameters for BP, BA, and IS.

Compound	Charge	Retention time/min^−1^	Parent ion	Daughter ion	Collision energy/eV^−1^
IS	Positive	1.50	417.38	296.90	45
BP	Negative	2.81	583.18	165.05	25
BA	Positive	2.91	607.18	589.10	25

**Table 2:** Regression equation and correlation coefficients for BP and BA (*y* = peak area ratio of BP or BA versus IS; *x* = concentration of BP or BA).

Compound	Linear range/ng mL^−1^	Regression equation	Correlation coefficient
BP	1–1000	*y* = 0.0996*x* + 0.2805	0.9998
BA	1–1000	*y* = 0.0985*x* − 0.2017	0.9982

**Table 3:** Precision, accuracy, recovery, and matrix effect of BP and BA in QC samples (*n* = 6).

Compound	Concentration/ng mL^−1^	Intraday precision and accuracy			Interday precision and accuracy			Recovery, %	Matrix effect, %
		Measured concentration/ng mL^−1^	RSD, %	RE, %	Measured concentration/ng mL^−1^	RSD, %	RE, %		
BP	1	0.99 ± 0.12	12.48	0.83	0.94 ± 0.08	8.53	5.50	99 ± 6.62	98 ± 11.20
	2	2.02 ± 0.12	6.00	−1.25	2.16 ± 0.07	3.64	−8.00	98 ± 8.31	99 ± 10.96
	500	505.16 ± 11.47	2.27	−1.03	524 ± 30.28	5.77	−4.80	90 ± 10.20	94 ± 3.27
	750	737.66 ± 23.72	3.21	1.64	750.16 ± 15.27	2.03	−0.02	93 ± 14.29	98 ± 7.83

BA	1	0.98 ± 0.08	8.40	1.66	0.89 ± 0.03	3.96	10.33	97 ± 7.88	99 ± 9.23
	2	2.11 ± 0.17	8.12	−5.83	1.93 ± 0.07	3.74	3.16	98 ± 5.52	96 ± 11.46
	500	494.83 ± 24.47	4.94	1.03	494 ± 15.65	3.16	1.20	97 ± 3.46	96 ± 8.58
	750	753.16 ± 18.25	2.42	−0.42	760.66 ± 15.94	2.09	−1.42	99 ± 3.97	98 ± 11.75

**Table 4:** BP and BA stability in QC samples (*n* = 6).

Compound	Concentration/ng mL^−1^	Three freeze thaw cycles			Short-term for 4 h (25°C)			Long-term for 30 Days (25°C)		
		Mean ± SD	RSD, %	RE, %	Mean ± SD	RSD, %	RE, %	Mean ± SD	RSD, %	RE, %
BP	1	0.99 ± 0.03	3.77	0.16	0.91 ± 0.05	5.60	9.00	0.88 ± 0.09	10.99	11.50
	2	2.04 ± 0.18	9.14	−2.00	1.85 ± 0.07	3.91	7.33	1.81 ± 0.08	4.79	9.50
	400	382.33 ± 32.20	8.42	4.41	397.50 ± 9.18	2.30	0.62	382.66 ± 11.86	3.09	4.33
	750	747.50 ± 9.87	1.32	0.33	743.00 ± 15.15	2.03	0.93	740.50 ± 4.18	0.56	1.26

BA	1	0.94 ± 0.06	6.38	6.00	0.93 ± 0.03	3.90	7.00	0.91 ± 0.04	4.86	9.00
	2	1.96 ± 0.10	5.27	1.91	2.00 ± 0.07	3.69	−0.41	2.19 ± 0.18	8.24	−9.83
	400	404 ± 8.09	2.00	−1.00	397.66 ± 16.04	4.03	0.58	396.83 ± 16.19	4.08	0.79
	750	746 ± 5.17	0.69	0.53	755.83 ± 13.99	1.85	−0.77	757.66 ± 17.71	2.33	−1.02

**Table 5:** Pharmacokinetic parameters of BP and BA in rats after intragastric administration.

Parameters	Unit	Values	
		BP	BA
*t*_1/2_^*∗*^	h	1.97 ± 0.23	1.47 ± 0.30
AUC (0–∞)^*∗*^	h ng mL^−1^	102.87 ± 31.74	65.81 ± 15.24
MRT (0–∞)^*∗*^	h	2.27 ± 0.32	2.01 ± 0.15
*T*_max_^*∗*^	h	0.35 ± 0.07	0.31 ± 0.03
Cl^*∗*^	h	1.15 ± 0.34	1.74 ± 0.36
*V*_*d*_^*∗*^	L/h/kg	3.71 ± 0.35	3.79 ± 1.47
*C*_max_^*∗*^	ng/mL	57.23 ± 17.18	49.76 ± 3.51

^*∗*^ *t*_1/2_: half-life for terminal elimination phase; AUC: area under the time-concentration curve; MRT: mean residence time; *T*_max_: time to *C*_max_; Cl: total plasma clearance; *V*_*d*_: apparent volume of distribution; *C*_max_: maximum plasma concentration.
