# Supplementary figures and images for: Rapid Determination of Isomeric Benzoylpaeoniflorin and Benzoylalbiflorin in Rat Plasma by LC-MS/MS Method
Source: Int J Anal Chem. 2017 May 8;2017:1693464. doi: 10.1155/2017/1693464 (PMC5439074; doi:10.1155/2017/1693464)

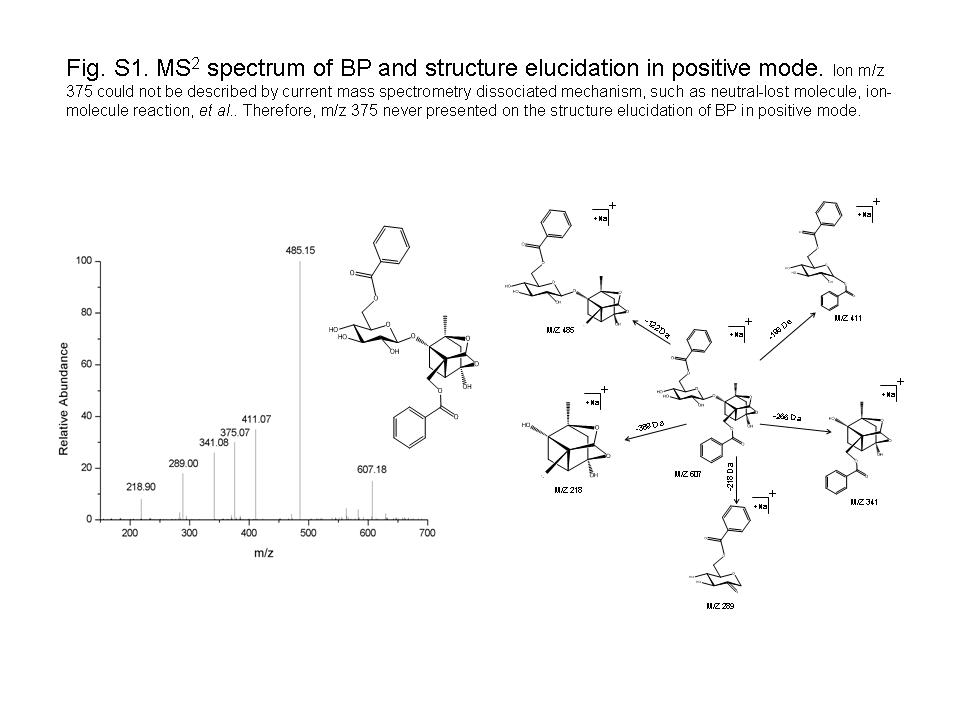

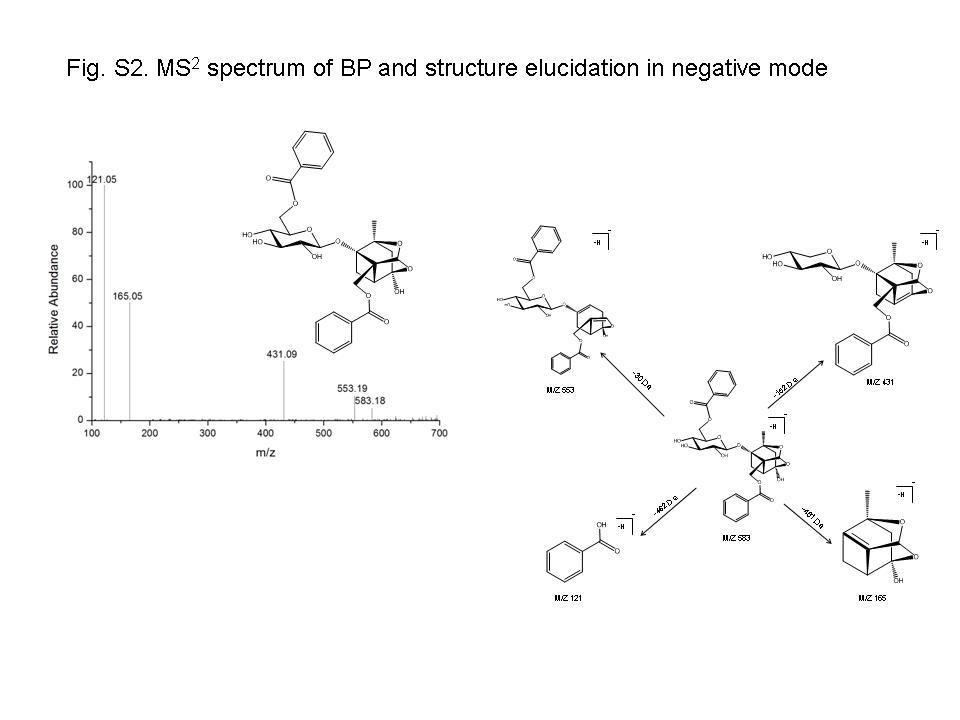

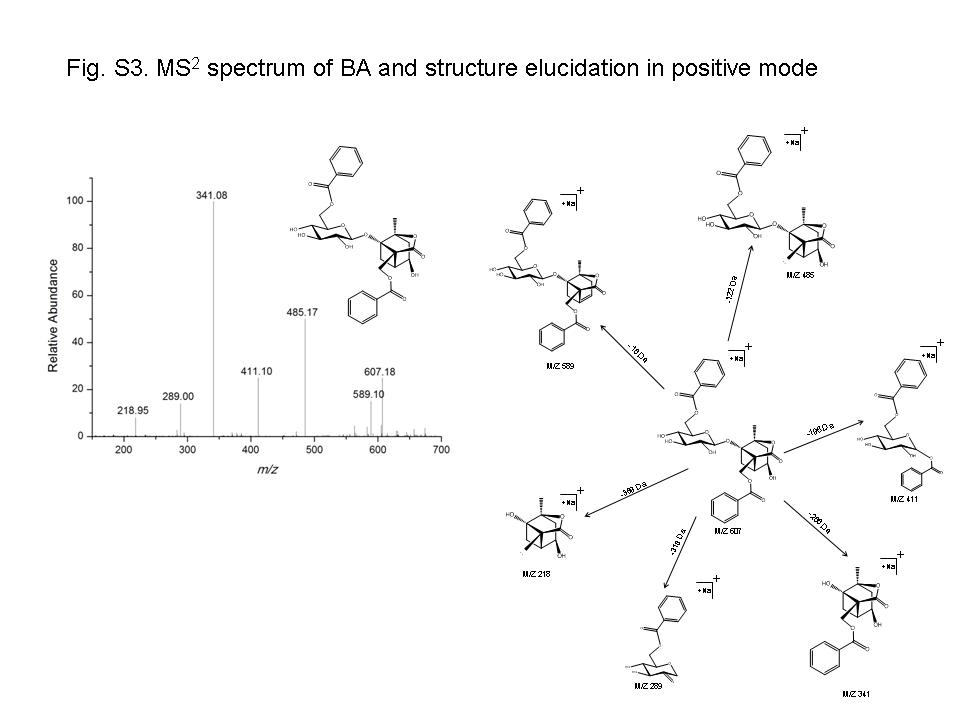

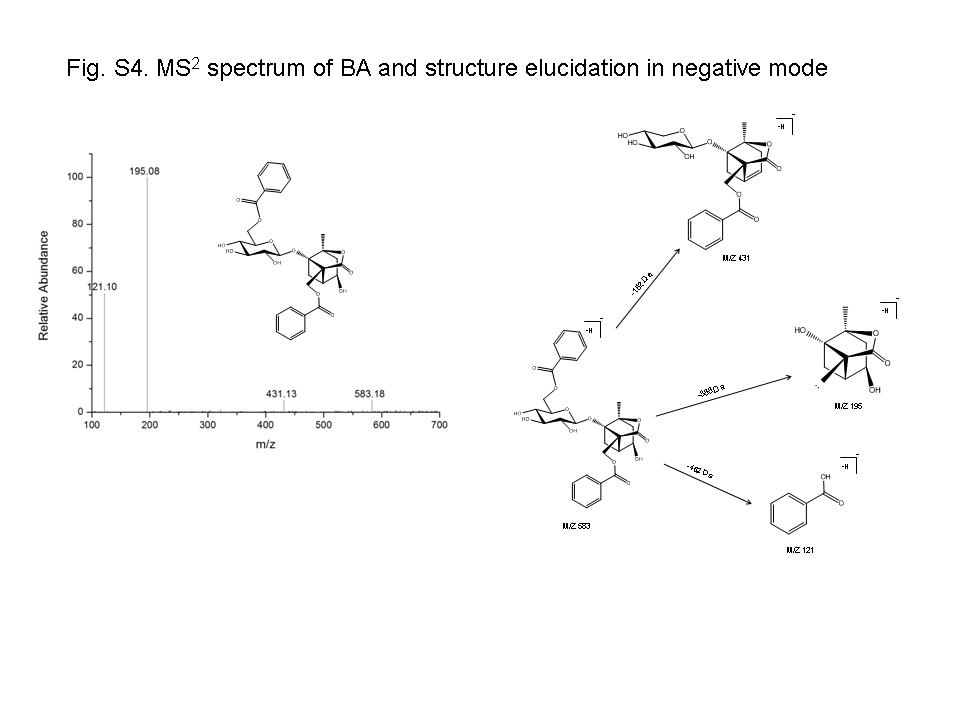

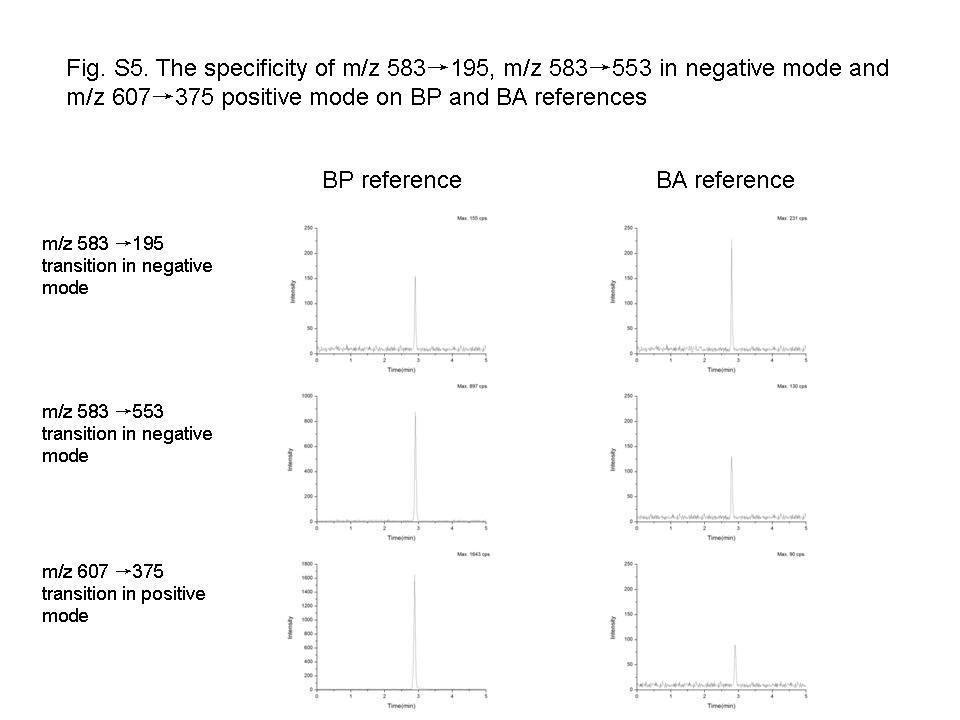

Supplement: Supplementary file 1 — Structure elucidations of BP and BA in positive or negative mode. [file 1693464.f1.doc]
